# Operative Techniques, Current Controversies, and Future Directions in the Surgical Management of Liver, Lung, and Peritoneal Metastases in Colorectal Cancer

**DOI:** 10.7759/cureus.96930

**Published:** 2025-11-15

**Authors:** Charlotte Cornwell, Pratik Raichurkar, Cherry Koh

**Affiliations:** 1 Colorectal Department, Royal Prince Alfred Hospital, Sydney, AUS; 2 Surgical Outcomes Research Centre (SOuRCe), Royal Prince Alfred Hospital, Sydney, AUS; 3 Institute of Academic Surgery (IAS), Royal Prince Alfred Hospital, Sydney, AUS; 4 Faculty of Medicine and Health, Central Clinical School, The University of Sydney, Sydney, AUS

**Keywords:** colorectal cancer liver metastases, colorectal lung metastases, colorectal peritoneal metastases, metastatic colorectal cancer, surgical management of metastatic disease

## Abstract

Colorectal cancer remains a substantial global health concern, with metastatic disease presenting an intimidating challenge. In recent decades, surgical management of metastatic disease to common sites, including liver, lung, and peritoneum, has advanced, reflecting improvements in surgical techniques, perioperative care, and multimodal treatment approaches. Historically, due to a dire prognosis, surgical interventions were reserved for symptom management and palliative intent. However, with the wider application of lung and liver resections to colorectal metastases as well as the development of cytoreductive surgery, outcomes for these patients continue to improve, and for some patients, the potential for cure. This narrative review will explore the operative techniques, current controversies, and future directions in the surgical management of liver, lung, and peritoneal metastases in colorectal cancer.

## Introduction and background

Colorectal cancer (CRC) is the fourth most prevalent cancer in Australia and is responsible for the second-highest number of cancer-related deaths, with 15,713 new cases and 5326 deaths in 2022 [1]. While patients with locoregional disease have an overall survival as high as 98% [2], approximately 18% of patients will have metastatic disease at the time of diagnosis. These patients suffer markedly poorer outcomes, with an estimated 5-year survival of 13%. Furthermore, a high proportion of patients with treated locoregional disease will go on to develop distant metastatic recurrence, reducing survival [2, 3].

The most common sites of metastatic involvement are liver (70%), lung (32% for colon cancer, 47% for rectal cancer), peritoneum (21% for colon cancer), bone (12% for rectal cancer) and nervous system (5% for colon cancer, 8% for rectal cancer) via hematogenous, lymphatic, contiguous and peritoneal routes. Patterns of metastatic disease vary with the anatomic site, history, and biochemistry of the primary tumour [4, 5]. While metastatic disease may be managed with a range of locoregional and systemic therapies, including radiotherapy, chemotherapy, ablation, and biologic therapy, the indications for surgical resection continue to expand. Resection plays a particularly important role in managing metastatic liver, lung, and peritoneal disease and, for some carefully selected patients, presents the potential for long-term disease-free survival or cure. In contrast, metastatic disease at other sites (brain, bone, soft tissue) is often not amenable to surgical resection. While hepatic metastasectomy is now well established, the role of surgery in pulmonary and peritoneal metastases remains more controversial, with ongoing debate regarding optimal patient selection, timing, and long-term benefit. Historically, surgery for metastatic CRC was considered largely palliative due to poor survival outcomes and limited systemic options. However, advances in imaging, perioperative care, and multidisciplinary team (MDT) decision-making have redefined patient selection and improved outcomes, establishing surgery as a key component of modern multimodal management. Despite advances in systemic therapy and surgical technique, uncertainty remains regarding patient selection, timing of resection, and the integration of surgery within multimodal treatment strategies. This review aims to critically evaluate operative strategies and controversies in the surgical management of liver, lung, and peritoneal metastases in colorectal cancer.

## Review

Methods

This narrative review summarises current evidence on the surgical management of CRC metastases to the liver, lung, and peritoneum. A structured search of PubMed, Embase, and relevant guideline documents was conducted for English-language studies up to 2025. Key outcomes, including overall survival, disease-free survival, recurrence, morbidity, and mortality, were extracted and presented as reported in the original publications, including p-values, 95% confidence intervals, and relevant comparators where available. No formal meta-analysis or statistical pooling was performed, and all results are cited directly from source studies to maintain transparency.

Liver metastases

Epidemiology and Clinical Context 

The liver is the most common site of metastatic disease in CRC, with up to 50% of patients developing metastases during the course of their illness [3]. This makes hepatic involvement a frequent and challenging clinical scenario. Surgery combined with systemic therapies now offers carefully selected patients the potential for long-term disease-free survival or cure. However, only about one-fifth of patients with colorectal liver metastases (CRLM) are considered eligible for potentially curative surgery, and resection is never offered for debulking purposes alone [3]. Importantly, up to 16% of patients initially deemed inoperable may later be rendered resectable after systemic therapy [4]. In contrast, patients with unresectable CRLM have a median survival of 10-12 months and negligible five-year survival, despite modern systemic regimens [5].

Indications and Resectability

The decision to proceed with liver resection is multifactorial, incorporating the number and size of metastases, adequacy of the future liver remnant (FLR), presence and control of extrahepatic disease, patient comorbidities, and response to neoadjuvant therapy. Guidelines from the British Association of Surgical Oncology (BASO) and the American Society of Colon and Rectal Surgeons (ASCRS) support a multidisciplinary, case-by-case approach [6-9]. The Fong Clinical Risk Score is often used to stratify recurrence risk, assigning points for node-positive primary tumour, disease-free interval <12 months, >1 metastasis, size >5 cm, and elevated CEA [8]. A summary of major randomised trials and cohort studies in liver metastases, alongside other metastatic sites, is provided in Table 1.

**Table 1:** Summary of major randomised and prospective studies evaluating surgical management of metastatic colorectal cancer RCT - randomized controlled trial; CRLM - colorectal liver metastases; HIPEC - hyperthermic intraperitoneal chemotherapy; CRS - cytoreductive surgery

Site	Key trial	Design	Intervention	Key findings	Citation
Liver	CAIRO5 (2023)	RCT	Modern chemo ± biologics for unresectable CRLM	Improved conversion to resection, higher PFS/OS	[26]
Liver	SECA-II (2018)	Cohort	Liver transplant for unresectable CRLM	Five-year OS ~80% in selected patients	[36]
Lung	PulMiCC (2020)	RCT	Pulmonary metastasectomy vs surveillance	No significant OS benefit (3.5 vs 3.8 yrs)	[43]
Peritoneum	PRODIGE 7 (2021)	RCT	CRS vs CRS+HIPEC (oxaliplatin)	No added benefit of HIPEC (median OS 41 months)	[47]
Peritoneum	COLOPEC (2022)	RCT	Prophylactic HIPEC in T4/perforated colon cancer	No reduction in recurrence	[48]

CRLM are broadly classified into resectable, unresectable, or borderline groups. Resectable disease requires that all metastases can be completely excised with preservation of two adjacent segments, adequate vascular inflow/outflow, and biliary drainage, and an adequate FLR [9]. Non-resectable disease typically involves >70% hepatic parenchyma, infiltration of both portal veins or all three hepatic veins, or metastases in >6 segments [10]. Borderline disease refers to potentially resectable cases complicated by technical or biological challenges, such as numerous lesions, disease progression, or extrahepatic involvement [11]. In such cases, adjuncts like portal vein embolisation (PVE), two-stage hepatectomy, or combined resection and ablation may convert patients to operability [12].

The absolute number of metastases is no longer considered a strict contraindication, with good outcomes reported even in patients with >3 lesions. Imaging plays a central role in assessment, with MRI outperforming PET/CT in sensitivity and specificity for CRLM detection [13].

Contraindications

Absolute contraindications to hepatic resection include an unresectable primary tumour, uncontrolled systemic disease, or extensive nodal metastases. Peritoneal disease was once considered incompatible with hepatic surgery, but selected patients undergoing combined cytoreductive surgery and hepatectomy have demonstrated encouraging survival outcomes, although long-term data remain limited [14].

Operative Principles

The principal aim of hepatic resection is R0 excision of all tumour while preserving adequate hepatic function. Resection can be anatomical (segmentectomy, lobectomy, or extended hepatectomy based on vascular segments) or non-anatomical (parenchymal wedge resections) [15]. Multiple studies confirm no significant difference in survival or recurrence between these approaches [15]. Increasingly, parenchymal-sparing resections are favoured, particularly for patients with chemotherapy-associated injury, recurrent disease, or limited hepatic reserve [15].

Advances in surgical technology have allowed laparoscopic and robotic resections for smaller, peripheral lesions. These approaches achieve similar oncologic outcomes to open resection, with reduced blood loss, pain, and hospital stay [16]. Major resections, however, are still most commonly performed via laparotomy.

Intraoperative blood loss strongly influences outcomes. Blood transfusion requirements are associated with increased recurrence and worse survival. To mitigate this, surgeons now employ advanced transection techniques (crushing, ultrasonic dissection, radiofrequency, water-jet, stapling devices) and vascular control strategies (Pringle manoeuvre, selective inflow control, total vascular exclusion) [15].

Resection Types

The choice of resection depends on tumour location and distribution. Anatomical resections include right or left hepatectomy, extended hepatectomy, and left lobectomy. Non-anatomical wedge resections are particularly useful for parenchymal preservation in patients with multifocal disease, underlying liver dysfunction, or recurrence. Both approaches are oncologically valid, though preservation of FLR is especially important in patients who have received neoadjuvant chemotherapy, as this can induce steatosis, sinusoidal obstruction, and impaired regeneration [15].

Timing of Surgery

Timing is straightforward in metachronous disease, typically requiring only coordination with perioperative chemotherapy [17]. Synchronous CRLM presents a greater challenge. These patients have worse survival than those with metachronous metastases (median OS 18.5 vs 62.5 months) [18]. Surgical strategies include:

Staged approach: Primary tumour resection (with or without neoadjuvant chemotherapy) followed by delayed hepatectomy. This risks progression of liver disease, especially if complications from primary resection delay systemic therapy (up to 30% of cases) [19].

Simultaneous approach: Combined colorectal and liver resection. Martin et al. reported this approach reduced complications and length of stay, while Reddy et al. showed worse outcomes when combined with major hepatectomies [19].

Liver-first (reverse) approach:* *Prioritises hepatic resection, particularly useful in patients with asymptomatic primary tumours and high liver burden. In de Jong's series, 22% developed subsequent primary obstruction, but most were managed with a diverting ostomy [20].

Recent high-level evidence supports systemic therapy to achieve resectability. The CAIRO5 trial (2023) demonstrated that modern chemotherapy regimens, including doublets and triplets with biologics, can downstage initially unresectable CRLM to operability, significantly improving progression-free and overall survival [21]. For an overview of prognostic scoring systems used in surgical selection across metastatic sites, see Table 2.

**Table 2:** Prognostic tools used in surgical decision-making for colorectal cancer metastases

Tool	Site	Variables	Prognostic Use	Citation
Fong Clinical Risk score	Liver	Node positivity, DFI <12 mo, >1 lesion, >5 cm, high CEA	Predicts recurrence after hepatectomy	[11]
Oslo score	Liver (transplant)	Lesion size, CEA, interval to transplant, disease stability	Identifies candidates for transplant	[33]
Peritoneal Cancer index	Peritoneum	13 regions scored by lesion size	Predicts survival; PCI >20 = poor outcome	[49]
Completeness of Cytoreduction score	Peritoneum	CC-0 = none, CC-1 = <2.5 mm, CC-2/3 = residual disease	CC-0/1 correlates with survival benefit	[50]

Liver Transplantation

Liver transplantation for CRLM was initially explored in the 1980s and 1990s, but was abandoned due to poor results: peri-operative mortality was high, 44% of grafts were lost, and five-year survival was only 18 % [22]. With advances in immunosuppression, patient selection, and perioperative care, interest has re-emerged.

The SECA I trial (2013) demonstrated that transplantation for unresectable CRLM was safe and achieved a five-year OS of 60%, though disease-free survival was limited (0% at two years, with recurrence common) [23]. The SECA II trial (2018), using stricter selection (stable disease on chemotherapy, low tumour load, normal/near-normal CEA), showed five-year OS approaching 80% [23].

More recently, centres in Milan and Toronto have confirmed that, in carefully chosen patients, survival outcomes after transplantation for CRLM approach those of hepatocellular carcinoma [24]. The Oslo Score, which incorporates the size of metastases, CEA level, interval from primary to transplant, and disease progression, provides a validated selection tool [23]. Controversy remains regarding donor allocation, given organ shortages, though strategies like living donation, extended criteria, and split grafts are under investigation. Tumour recurrence under immunosuppression is an additional unresolved concern [23].

Outcomes and Recurrence

Even after resection, recurrence is common, with up to 50% of patients relapsing within two years [25]. Repeat resection is feasible in selected cases and can provide survival benefits comparable to initial hepatectomy [25]. Perioperative morbidity following liver resection varies with extent and indication. In one high-volume cohort of 564 patients, complications occurred in 46% (Clavien-Dindo grade II in 30%, grade III in 15%, grade IV in 1%), with 90-day mortality of only 0.5% [25]. Major complications include post-hepatectomy liver failure, bile leak, intra-abdominal sepsis, and bleeding. Comparative outcomes of surgical resection across liver, lung, and peritoneal metastases are outlined in Table 3.

Lung metastases

Epidemiology and Clinical Context

The lung is the second most common site of distant spread in CRC, present in ≈15% of patients with systemic disease [26]. Isolated pulmonary metastases are less common, occurring in only ≈5% of patients, as lung disease frequently co-exists with hepatic involvement [26-28]. Pulmonary metastasectomy has been widely practised for decades across malignancies, including CRC, but its survival benefit remains controversial due to limited high-level evidence [29, 30].

Evidence for Pulmonary Metastasectomy

Retrospective series and institutional analyses suggest a survival advantage in selected patients. Five-year survival rates of 30-50% have been reported in resected cohorts, compared with <5% in patients managed without surgery [27, 28, 31]. Surgical mortality is low (0-2.5%), making it a relatively safe procedure [26].

Patient Selection and Prognostic Factors

Surgery is reserved for carefully selected patients where complete resection of all pulmonary disease is achievable with acceptable parenchymal preservation. Selection criteria include absence or control of extra-thoracic disease, adequate cardiopulmonary reserve, and a likelihood of achieving R0 resection [27].

Good prognostic factors include unilateral disease, long disease-free interval (>12 months), few metastases (<3), and low CEA [27-29]. Adverse prognostic features include elevated CEA, rapid doubling time (< 100 days), mediastinal nodal involvement, and uncontrolled primary or extra-pulmonary disease [27]. Node positivity strongly predicts poor outcome: five-year survival is ≈17% for hilar nodes and approaches 0% for mediastinal nodes [27].

Repeat resections are feasible in recurrent disease. Some series report survival outcomes similar to first resections, though results vary and are limited by selection bias [28, 29, 31]. Relevant prognostic tools used in metastatic disease selection are shown in Table 2.

Operative Approaches

The surgical objective is complete clearance of intrathoracic disease with preservation of lung tissue. Non-anatomical wedge resection is the most common procedure, performed in about two-thirds of CRC pulmonary metastasectomies [28]. Segmentectomy offers greater oncologic clearance with lower recurrence rates than wedge resection [29]. Lobectomy is usually reserved for larger or central tumours, while pneumonectomy is rarely performed due to high morbidity and limited oncologic gain [28].

Hilar and mediastinal lymph-node sampling or dissection is recommended, though its role is largely prognostic rather than therapeutic.

Minimally Invasive Versus Open Surgery

Open thoracotomy, once standard, allows bimanual palpation to detect occult lesions. However, video-assisted thoracoscopic surgery (VATS) has gained popularity, offering equivalent oncologic outcomes in selected cases with reduced morbidity, pain, and length of stay [28, 31].

The limitation of VATS is the reduced ability to detect occult disease. Up to 18% of patients with apparently solitary nodules on CT have multiple metastases detected intra-operatively by palpation [32, 33]. To overcome this, surgeons now use intraoperative adjuncts such as radiotracers, fluorescent dyes, hookwires, and virtual-assisted lung mapping [34].

Robotic-assisted thoracic surgery (RATS) is an emerging technique with enhanced dexterity and visualisation, though evidence remains limited. For bilateral disease, staged VATS or a median sternotomy may be considered; however, clamshell thoracotomy is rarely used due to morbidity [30, 31, 35].

Perioperative Morbidity and Complications

Pulmonary metastasectomy is generally safe. Reported complication rates are 3-10%, with VATS associated with fewer complications than thoracotomy [31]. Common issues include prolonged air leak, bleeding, pneumonia, and wound infection. Rarely, disease recurrence has been documented at VATS port sites [36]. Mortality is consistently <2.5% [26].

Outcomes and Controversies

Despite widespread use, the survival benefit of pulmonary metastasectomy remains unproven at the highest level of evidence [30]. Retrospective and registry studies report improved survival compared with non-operative cohorts, but these are prone to selection bias. The PulMiCC trial raised important doubts, suggesting that surgical benefit may be overstated and that tumour biology is a stronger determinant of outcome than resection itself [29].

Nevertheless, pulmonary metastasectomy remains common practice in high-volume centres, underpinned by the principle of complete macroscopic clearance in oligometastatic disease. Alternatives for non-surgical candidates include stereotactic body radiotherapy (SBRT), radiofrequency ablation, or systemic therapy, though these are generally palliative and lack equivalent survival outcomes [31, 33]. Comparative long-term survival and recurrence data across sites are shown in Table 3.

**Table 3:** Comparative outcomes and complications of surgery by metastatic site

Site	Median OS after surgery	5-year survival	Recurrence patterns	Key complications
Liver (resection)	40-60 months	40-58%	Intrahepatic ± lung	Bile leak, liver failure
Liver (transplant, SECA-II)	60-80 months	~80% (selected)	Nearly all recur, often resectable	Immunosuppression, graft loss
Lung	30-40 months	30-50% (selected)	Contralateral lung, systemic	Prolonged air leak, pneumonia
Peritoneum (CRS-HIPEC)	22-40 months	~20% at 10 years	Peritoneum, liver	Leak, sepsis, ileus

Peritoneal metastases

Epidemiology and Clinical Context

Peritoneal metastases occur in about 5% of patients at initial CRC diagnosis and represent 25% of synchronous metastases [37]. Prognosis is poor, with untreated survival typically five to nine months [38, 39]. Historically, peritoneal involvement was considered terminal, with limited treatment options. However, since the 1990s, cytoreductive surgery (CRS) combined with hyperthermic intraperitoneal chemotherapy (HIPEC), pioneered by Paul Sugarbaker, has transformed management strategies [40, 41].

Cytoreductive Surgery and HIPEC

CRS aims to remove all visible peritoneal disease, using peritonectomy procedures and resection of affected viscera [42]. HIPEC delivers heated chemotherapy intra-operatively to treat microscopic residual disease. Early studies suggested significant survival improvements compared with systemic therapy alone, establishing CRS-HIPEC as the standard for selected patients [43-46]. Multiple RCTs reported median survival of ≈22 months with CRS-HIPEC compared to ≈12 months with systemic chemotherapy alone [43-46].

However, enthusiasm for HIPEC has been tempered by more recent trials. The PRODIGE-7 trial (2021) demonstrated that while CRS significantly improved survival, the addition of oxaliplatin-based HIPEC did not provide additional benefit (median OS 41.7 months CRS vs 41.2 months CRS-HIPEC) [47]. These findings challenge the necessity of HIPEC in standard protocols and shift focus back to the quality of cytoreduction as the primary determinant of outcome.

The COLOPEC trial (2022) further questioned HIPEC's role. This RCT evaluated prophylactic HIPEC in high-risk patients with T4 or perforated colon cancer. HIPEC did not reduce the rate of peritoneal recurrence compared with standard follow-up (18% vs 18% at 18 months) [48]. Together, PRODIGE-7 and COLOPEC indicate that HIPEC may not provide the additional survival advantage once believed, at least with oxaliplatin. These pivotal trials are summarised in Table 1.

Patient Selection and Prognostic Tools

Careful selection is critical, given the morbidity of CRS. Patient factors (age, comorbidity, cardiopulmonary reserve) and tumour biology (histology, molecular profile) are essential considerations.

The Peritoneal Cancer Index (PCI) remains the most widely used tool to quantify disease burden. It divides the abdomen into 13 regions and scores each for lesion size, generating a total from 0-39. Higher PCI scores predict poorer outcomes, and surgery is rarely offered for PCI >17-20 [49].

The Completeness of Cytoreduction (CC) Score measures residual disease after CRS: CC-0 (no visible disease) and CC-1 (<2.5 mm nodules) are associated with improved survival, whereas CC-2 and CC-3 reflect incomplete cytoreduction and poor prognosis [50]. CRS is unlikely to benefit patients in whom complete cytoreduction cannot be achieved. The PCI and CC scores, alongside other prognostic indices, are summarised in Table 2.

Imaging and Preoperative Assessment

Accurate pre-operative staging is vital, but imaging modalities remain limited. CT and PET-CT have low sensitivity for small-volume disease, particularly nodules <5 mm. Diffusion-weighted MRI is the preferred modality for detecting peritoneal deposits [49]. Diagnostic laparoscopy often supplements imaging, providing direct assessment and biopsy, though it cannot visualise all peritoneal compartments.

Contraindications

Absolute contraindications to CRS-HIPEC include extensive extra-abdominal metastases, uncontrolled systemic disease, and bulky retroperitoneal nodal involvement. Peritoneal disease associated with bowel perforation or obstruction is not an absolute contraindication, but these features predict poorer outcomes [49]. Combined liver and peritoneal resections can be performed in selected patients, with growing evidence of feasibility and potential survival benefit [14, 51].

Operative Techniques

CRS was pioneered by Sugarbaker in the 1990s, and despite ongoing evolution since then, the principles remain mostly unchanged [40, 42]. CRS is performed via a midline incision from the xiphoid to the pubis, excising the umbilicus and previous scars, including port sites if present. PCI should be calculated upon entering the abdomen to confirm suitability to proceed with CRS. Inspection of the abdominal cavity in combination with pre-operative imaging and findings at diagnostic laparoscopy determines the exact operative approach, which varies between patients. Peritonectomy procedures include anterolateral parietal peritonectomies, right and left sub-diaphragmatic peritonectomies, stripping of Glisson's capsule, and pelvic peritonectomies. Greater omentectomy is performed in most cases. The small bowel and its mesentery are often involved, and disease here can be addressed with high-powered electrocautery or resection if required. Diaphragm, colon, small bowel, rectum, stomach, bladder, spleen, gallbladder, pancreas, and liver resections are performed if involved with anastomoses and reconstruction as required. Morbidity increases with the number of bowel anastomoses and peritonectomy procedures performed.

Morbidity and Mortality

CRS-HIPEC is associated with high morbidity, reported at 40-60 %. Major complications include bleeding, anastomotic leak, intra-abdominal abscess, sepsis, ileus, and pleural effusion [52]. Mortality ranges from 0.9-5.8 % depending on patient selection and centre experience [52]. The risk of complications increases with higher PCI and a greater extent of cytoreduction.

Recurrence and Repeat Surgery

Despite aggressive management, recurrence is common. About 70 % of patients relapse within two years, often with peritoneal or hepatic recurrence [49]. Risk factors include synchronous peritoneal disease, T4 primaries, proximal colon location, BRAF mutations, and high-grade histology.

Repeat CRS-HIPEC is increasingly performed in selected patients, with survival outcomes similar to initial procedures and without increased morbidity or mortality [53, 54]. In one large series, two-year survival after repeat CRS was 39% for CC-1 resections compared with 5% for CC-3 resections [50].

Emerging and Alternative Therapies

The limitations of HIPEC have prompted investigation of alternative intraperitoneal therapies. Pressurised intraperitoneal aerosol chemotherapy (PIPAC) allows laparoscopic delivery of chemotherapy as an aerosol under pressure, enhancing penetration. Early studies suggest safety and feasibility, with ongoing trials assessing efficacy [55].

Molecular stratification is also under investigation. Mutational profiles such as BRAF, RAS, and microsatellite status influence recurrence and survival and may guide patient selection in the future [56].

Outcomes and Current Controversies

For well-selected patients, CRS offers a significant survival benefit over systemic therapy alone, with median OS exceeding 40 months in contemporary series [47]. The main controversies now relate to the role of HIPEC. PRODIGE-7 and COLOPEC strongly suggest that oxaliplatin-based HIPEC adds no additional survival advantage beyond CRS alone [47, 48]. Whether alternative agents or delivery methods may prove effective remains an area of active research.

Despite advances, long-term outcomes remain modest. Ten-year survival after CRS is ≈20%, and most patients ultimately experience recurrence [49]. The challenge is therefore to balance potential survival benefits against high morbidity and the significant physiological demands of surgery. Table 3 compares outcomes and complications of surgery across metastatic sites.

## Conclusions

The management of colorectal cancer with liver, lung, and peritoneal metastases continues to advance, with surgery remaining central in carefully selected patients. Optimal outcomes depend on early identification, individualised treatment planning, and a multidisciplinary approach. While prognosis remains guarded, ongoing innovation in surgical and systemic strategies offers the potential to improve survival and quality of life.
